# Quantification and Analysis of Offensive Situations in Different Formats of Sided Games In Soccer

**DOI:** 10.2478/hukin-2014-0125

**Published:** 2014-12-30

**Authors:** Jorge Diaz-Cidoncha Garcia, Ignacio Refoyo Román, Julio Calleja-González, Alexandre Dellal

**Affiliations:** 1 Fédération Internationale de Football Association (FIFA) / Education and Technical Development Department.; 2 National Institute of Physical Education (INEF), Polytechnic University of Madrid / Sports Department.; 3 National Institute of Physical Education (INEF), University of Basque Country.; 4 FIFA Medical Excellence Centre, Santy Orthopedicae clinical, sport science and research department, Lyon, France.; 5 Unité de recherche de l’OGC Nice (soccer), Nice, France.

**Keywords:** game format, soccer, attacking move, technique, tactics

## Abstract

There has been a lot of research that enabled soccer to improve: its technique, tactics and strategy through analysis and training. Nevertheless, players’ need to interact with each other turns any defending or attacking situation into complex solutions with a wide range of variables to be considered, in which the player is never isolated and must make the move that has the most positive impact on play. Fifty-four sided games played in three different formats (5v5, 7v7 and 9v9) and with two age groups (U9 and U14) were filmed at three soccer clubs in Spain in order to identify the most relevant attacking moves, from a technical and tactical perspective. This study used the observational method; it is descriptive and is applied through well-prepared systematic quantitative observation in a natural environment. A key part of the method involved viewing the match recordings and logging moves that had been categorised beforehand. Cohen’s Kappa analysis showed that the results for the most representative variables presented a substantial degree of concordance (0.61–0.80). The results show that there were significant variations depending on the game format, and the following study will present a description and analysis of the aspects that had considerable influence on attacking moves in different formats of sided games (5v5, 7v7 and 9v9). The study also presents various practical applications for the area of training and analysing both youth and professional soccer.

## Introduction

Over the course of the past decade, playing soccer in reduced spaces has become particularly important, both for organised or spontaneous set-ups (Castellano et al., 2008). This type of play offers a great deal of possibilities and combinations and gives participants an increased level of interaction in the game (McGarry et al., 2002). Sided games (SG) are very beneficial for players, particularly during learning stages of grassroots and youth football (Castellano et al., 2011) and for training senior players. By SG we mean 5v5, 7v7 and 9v9 formats using the goal or not. Consequently, the pitch dimensions are smaller than in an 11v11 format and the rules are adapted to each format (e.g. the goals and the boxes are smaller). The study and analysis of match situations, particularly those where players interact with each other, are of great importance in that they identify the factors that influence correct decision making which may lead to success in the game of football (Gréhaigne et al., 2011). This undertaking requires a particularly complex implementation due to a large number of variables that are influential in a single team play. All players (both from the team itself and the opposing team) directly impact the final result of play, depending on what they do and the decisions they make.

There are several aspects which give rise to this complexity in the analysis of small-sided games: a high number of players involved in the development of play, interactive nature of the players’ moves, a degree of evolution and the “internal logic” of soccer, a high number of direct and indirect performance factors and the pitch size determined from the competition itself. Experience (Di Salvo et al., 2009; Kelly and Drust, 2008) has shown that players get more touches of the ball, learn quicker and take more decisions during the game (player concentration increases because the ball is never far away). There will also be a greater degree of participation as there are fewer players on the pitch and therefore increased individual attention from the participants is guaranteed. It is worth noting that because players are continually exposed in SG, there will be more attacking and defending situations. FIFA (2012) presents statistics to explain some of the differences among various small-sided formats, enabling us to compare the number of moves that take place during a play: players touch the ball five times more often in 4-a-side soccer and 50% more in 7-a-side soccer; players are three times more often in one-against-one situations in 4-aside soccer and twice as often in 7-a-side; goals are scored every two minutes in 4-a-side soccer on average and every 4 minutes in 7-a-side; goalkeepers are involved in the action two to four times more often in 7-a-side soccer than in 11-aside soccer; and, the ball is out of play 8% of the time in 4-a-side soccer, 14% in 7-a-side and 34% in 11-a-side soccer.

### Tactical and strategic moves in football in reduced spaces

For this study a move is understood as a significant combination, more or less complex, of various motor and mental processes, which are indispensable to solve a problem arising from a game situation. Tactics are characterised as an intelligent combination of motor resources, individual and collective, to solve game situations as they occur as a result of the competitive activity itself, and also as the decisions, taken before the game, on the choice and order of moves. Strategy shows how to set up opportunities that should be exploited tactically (Gabbet and Mulvey, 2008).

## Material and Methods

### Participants

A cross section study consisting of fifty-four grassroots soccer games from the U-9 and U-14 age male groups was conducted. The physical characteristics of the players were as follows: for U-9, body height 134.1 ± 12.3 cm; body mass 29.4 ± 11.6 kg and for U-14, body height 163.0 ± 13.8 cm; body mass 52.9 ± 13.1 kg); it is remarkable that there are no recent studies that compared the variables of this study for the U-9 and U-14 age groups. The same teams were monitored for a specific period of time to ensure that the cross section was as broad as possible. Furthermore, all of the formats (5v5, 7v7 and 9v9) were considered when selecting the cross section. The games were recorded at three different clubs: the football academy of the RFEF (Spanish Football Association), Adarve-Barrio del Pilar and Villanueva del Pardillo (all of them playing in the first or second state youth leagues). Of the 54 games in the cross section, the following recordings (and subsequent analyses) were made: 18 in each club (RFEF, Adarve and Villanueva del Pardillo), 18 on each playing surface (natural turf, artificial turf, soil), 18 in each format (5v5, 7v7, 9v9), 36 for each age group (U-9 and U-14). Each game lasted 20 minutes, with no breaks and no substitutions. A cross section with high representativeness and which covers all of the possible parameters that influence the development of the game was chosen. The Madrid Polytechnic University’s Institutional Review Board approved the use of human subjects in this research for the purpose of collecting the data and statistically analysing it, according to the declaration of Helsinki.

### Equipment – instruments

The equipment used for organising the SG consisted of balls (size 4 for U-9 and size 5 for U-14), different coloured bibs, cones and markers, mini goals and seven-a-side goals (depending on the game format). The games were timed using a Traceable digital stopwatch. The pitch measurements were 20 × 30 m for 5v5, 30 × 45 m for 7v7 and 45 × 60 m for 9v9. The games were recorded using a Sony HDR-CX570 camera and a HI-POD tripod, which were acquired by the FIFA’s Education and Technical Development Department and can continue to be used for this study in the next coming months, should this be necessary. The games were watched and analysed on a TV monitor once all of the scheduled sessions had been recorded and categorised properly. Each player had a pitch radio of 4.8 m for 5v5, 6.4 m for 7v7 and 5.5 m for 9v9.

### Measures

There are currently very few studies that reflect the tactical and technical differences among various formats of SG (5v5, 7v7 and 9v9) and different playing surfaces. For this reason, technical and tactical variables were recorded for each of the games analysed and then compared against each other so as to reflect their main characteristics and the differences among them. Therefore, just one team within a club and in the same age group was monitored so that the players and teams analysed would be the same and the nature of the study would not vary from one recording to the next. Furthermore, an initial assessment was applied before each game to ensure that the conditions relating to size and quality of the playing field, climate, and support facilities for the AV recording, etc. were acceptable; external conditions were standardized (24º external temperature, 60% humidity).

### Procedures

The videos of the 54 games used for this study were recorded on the soccer pitch. All of the games were watched on site and then analysed from the recordings, with the same process being followed at all times, which was carried out by a minimum of three different reviewers. The data was collected as the recordings were watched, with previously defined variables being monitored. The study was conducted using the observational method; it is a descriptive study and was carried out by way of observation that was systematised, prepared beforehand, carried out in a natural setting and of a quantitative nature. The observational method was applied as follows: formulation of a problem, collection and recording of data, analysis and interpretation of the observed data and communication of the results.

### Statistical Analyses

After applying a normality test, the arithmetic average of each and every variable previously recorded from the footage of the 54 games was calculated. For the percentage variables (time and pitch zones), the percentages for each of them were compared. A Pearson’s chi-squared test was performed with a level of significance (Pearson’s X^2^ <.05). Cohen’s Kappa has been analysed for the variables of the ball out play, touches per game and attempts at goal. Standard deviations were also calculated in every figure. SPSS 22.0 (Chicago, Illinois, USA) pack was used for every statistic calculation.

## Results

**Figure 1:**
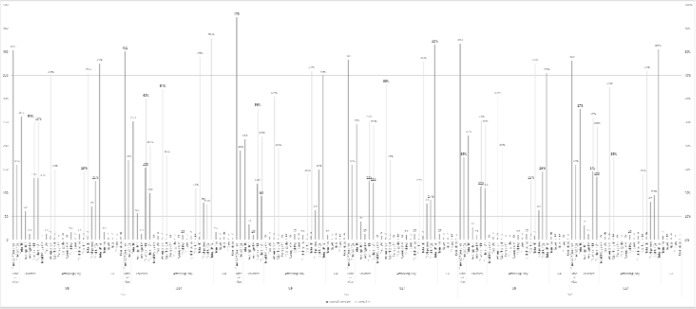
Summary of the technical variables by age and game forma BOP = ball out of play; TG = touches per game; TOP = touches per outfield player; TM = touches per minute; DH = defensive half; AH = attacking half; GK = goalkeeper; AG = attempts at goal; SP = shots per minute; GM = goals per minute; PAE = penalty area entries; UD = unsuccessful dribbles; SD = successful dribbles; UP = unsuccessful passes; SP = successful passes.

The averages of all variables are presented below as well as the percentages for the ball being out of play and for play in a specific half of the pitch (attacking or defensive). All of the results are arranged according to the game format, age group and club from which they were collected. Figure 1 gives an overall summary of all of the values for each age group and game format. Significant differences can be noted in case of the number of touches for the players per game, including goalkeepers.

**Figure 2:**
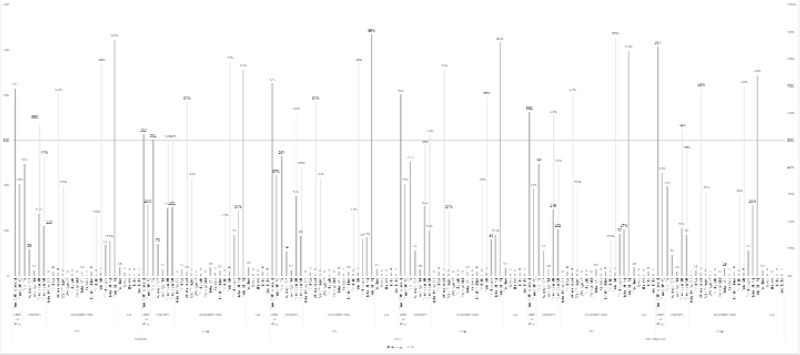
Results of the variables for 5v5. U9 and U14 BOP = ball out of play; TG = touches per game; TOP = touches per outfield player; TM = touches per minute; DH = defensive half; AH = attacking half; GK = goalkeeper; AG = attempts at goal; SP = shots per minute; GM = goals per minute; PAE = penalty area entries; UD = unsuccessful dribbles; SD = successful dribbles; UP = unsuccessful passes; SP = successful passes

The results in Figure 2 show that the total number of touches per game (263 for U-9 and 253 for U-14) as well as the average number of touches per outfield player (62 and 56 for each age group, respectively) were significantly higher in 5v5 format than in the other game formats. The same for touches in the defensive and attacking halves, where high values were recorded in both halves (142.5 touches of the ball in the defensive half and 115.5 in the attacking half); the distribution of touches was similar, where values close to 50% were recorded in both: attacking half and the defensive half. The results also show that the total number of attempts at scoring was higher in the 5v5 format (13 for U-9 and 8 for U-14, with 67% on target). The number of times the ball entered the penalty area and the attempted dribbles also shows that there were more attacking moves in the 5v5 format, with the ball penetrating the area 17 times and 13 attempted dribbles in the U-9 age group. The number of attempted passes – both successful and unsuccessful – was also higher in the 5v5 format, where 75% and 86% of the passes were successful for the U-9 and U-14 age groups, respectively. In comparison with the other formats, the goalkeepers showed higher values in all moves recorded in the 5v5 format, with a total average of 17 touches, 5 saves, 5.5 kicks, 3 throws and 5.5 goal kicks per match.

**Figure 3:**
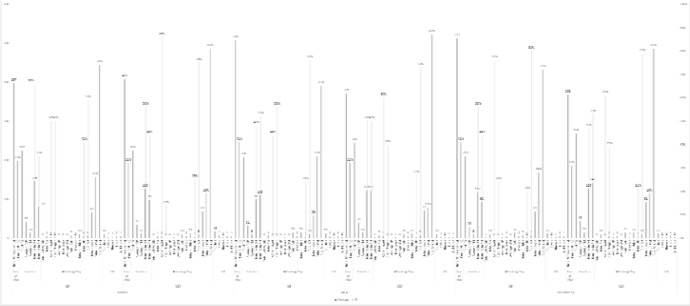
Results of the variables for 7v7, U9 and U14 BOP = ball out of play; TG = touches per game; TOP = touches per outfield player; TM = touches per minute; DH = defensive half; AH = attacking half; GK = goalkeeper; AG = attempts at goal; SP = shots per minute; GM = goals per minute; PAE = penalty area entries; UD = unsuccessful dribbles; SD = successful dribbles; UP = unsuccessful passes; SP = successful passes

Figure 3 shows that the ball was out of play longer in the 7v7 format, where it was stopped for 38% of the time in the U-9 age group and 32% of the time in the U-14. Despite being lower than in the 5v5 format, the number of touches among the outfield players in the 7v7 format was higher than in the 9v9 format (average of 33 and 39 touches for each age group, respectively). The number of touches in the defensive half was higher in 7v7 than in the other two formats and accounted for 56% of the touches in the U-9s and 51% in the U-14s. However, the number of touches in the attacking half was lower than in the other two formats (44% and 49%, respectively). Seen against the total number of attempted passes, unsuccessful passes were also quite high, accounting for 30% of the attempted passes in the U-9 age group and 17% in the U-14s.

**Figure 4:**
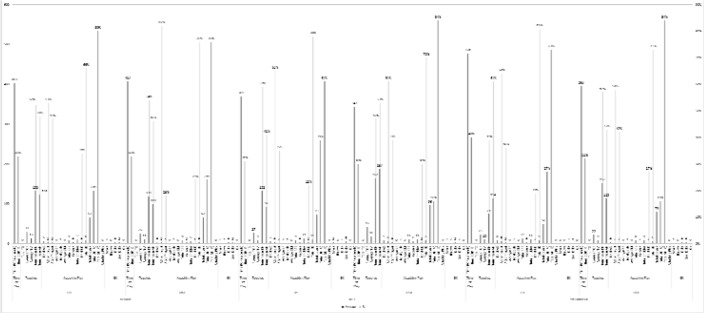
Results of the variables for 9v9. U9 and U14 BOP = ball out of play; TG = touches per game; TOP = touches per outfield player; TM = touches per minute; DH = defensive half; AH = attacking half; GK = goalkeeper; AG = attempts at goal; SP = shots per minute; GM = goals per minute; PAE = penalty area entries; UD = unsuccessful dribbles; SD = successful dribbles; UP = unsuccessful passes; SP = successful passes

As shown in Figure 4, the ball was out of play for a considerable amount of time in the 9v9 format, specifically 35% of the time in the U-9 age group and 32% of the time in the U-14s. The total number of touches per match was high, reaching a total of 278 touches in the U-14s. Of all formats, however, the average number of touches per outfield player was lowest in the 9v9 format (29 touches per player per match). The ball was in play in both halves of the pitch for an almost equal amount of time, with 50.5% of the actions of play being recorded for the defensive half and 49.5% for the attacking half. The number of off-target shots was highest in the 9v9 format, where 396 off-target shots were recorded in the U-9 age group and 35% of the shots were off target in the U-14s. The figures recorded for the attacking play in 9v9 were similar to those in the 7v7 format and significantly lower than in 5v5. The goalkeepers also participated less in the play in 9v9 than in the other two formats and had a total of 7.5 touches per game, 2 saves, 3 kicks and 0.5 goal kicks per game.

The results of the chi-squared test performed on the technical and tactical variables collected during the games are also considered in this study. A level of significance of alpha 0.005 was chosen and the associated table value for χ^2^ to two degrees of freedom and alpha 0.05 was therefore 5.99. The hypothesis stated that the frequency of moves depended the on game format. Therefore, given that there were three different formats, one third of the moves was expected to occur in each format (5v5, 7v7 and 9v9). Given that the probability was lower than alpha (the values were on the left in the goodness of the fit chart), it could be concluded that the hypothesis was correct and that the frequency of the moves would therefore depend on game format.

## Discussion

The study showed that there were more touches of the ball and attacking play in the smaller game formats (Sampaio and Maças, 2012); there was a higher frequency among the variables for attacking play in all age groups and playing surfaces (goals, shots on goal and balls entering the penalty area) in the smaller-sided games (5v5 and 7v7) than in the 9v9 format (Okihara et al., 2004). It should be noted in particular, that the U-9 age group playing in the 5v5 format created more attacking moves and attacks than any other age group and game format, directly influencing the intensity of play (Koklu et al., 2011) and therefore, the learning process during training (Iaia et al., 2009). Furthermore, the main trend when it comes to the number of touches of the ball for outfield players across all age groups and playing surfaces was that the number of touches was significantly higher in 5v5 and 7v7 than in 9v9 (Lago and Martín, 2007). The same trend could be observed for the goalkeepers (Di Salvo et al., 2007), with them having a higher number of touches in 5v5 and 7v7 than in 9v9 for all playing surfaces and in both age groups. On the whole, by comparing the levels of attacking play in each format, we can see that the general trend was for the frequency of all variables of attacking play to be higher in the smaller-sided games (5v5 and 7v7) than in the 9v9 format (Jones and Drust, 2007; Tenga et al., 2010).

With regard to playing time and possession, the most obvious trend across the different types of playing surface was that on artificial turf, the ball was out of play considerably longer in the U-9 age group (41%) than in the U-14 age group (32%). For the U-9 age group, the ball was out of play for less time on a natural grass surface (37% for 5v5, 32% for 7v7 and 30% for 9v9). Age and physicality had a significant influence on the variables that corresponded to the players’ technical, tactical and physical performances (Silvestre et al., 2006); even though the trends listed above were consistent in most of the formats and age groups, we found variations in the technical variables across the various age groups (U-9 and U-14). The ball was out of play in the U-9 age group for less time in the 5v5 format and for more time in the 7v7 and 9v9 formats, making play more broken and directly influencing its intensity (Dellal et al., 2008). In the U-14 age group, however, the ball was out of play for less time in the 9v9 format and for more time in 7v7. With respect to attempted passes in both the 7v7 and 9v9 formats, the U-14 age group tried more passes than the U-9 group, thus following what they had previously learned as well as the demands of the game format (Dellal et al., 2011a). Furthermore, there was a much higher percentage of success across the data collected in the U-14 age group than in the U-9 (87% compared to 64% in 7v7, and 83% compared to 73% in 9v9). Although, there was a clear tendency towards greater levels of attacking play in the 5v5 format than in 9v9 for both age groups (Hill-Haas et al., 2009), there were more attacking moves in the 9v9 format among the U-14 players. The variations in the technical variables between the two age groups can be explained by the fact that the U-14 age group is technically more competent than the U-9 group and is more used to competitive situations (Dellal et al., 2011b). Furthermore, the data collected from the U-14 age group show more positive values for surfaces that facilitate passing and building attacking play, such as artificial and natural turf.

Having discussed the most representative values for the technical and tactical moves reflected in different variables of this study, it can be said that all of them are closely interrelated (Myers et al., 2004) and change uniformly depending on the type of the playing surface, game format and age group. Therefore, they cannot be seen as isolated moves but rather as part of a co-evolution. One of the most important goals of any coach is to get his players to develop a high level of technical and tactical ability, because in most cases, it is not the competitor with the most stamina, strength, speed or flexibility who wins, and neither is it the player who is able to give the best technical delivery in terms of biomechanics, but rather it is the athlete who is able to perceive a variety of different situations that occur during a match, analyse them correctly and make the right move technically, assessing his own situation against that of his rival.

## Practical applications

The results of this study show the relevance of reduced spaces in learning how to play football and in training sessions. Players are involved in more decision-making and moves in small-sided formats, which results in a greater volume of these in practice (both in learning and training), in the technical and tactical variables as well as in the physical variables (Reilly and White, 2004). In small-sided soccer, playing systems are not particularly important, especially at an early age. Nevertheless (and bearing in mind the theoretical terms defined above), it can be said that the concepts of tactics and strategy gain special importance in soccer played in reduced spaces given the large number of moves in which players are involved (Leser et al., 2011). All of the factors that influence moves, such as the surface, the number of players and their ability, will quickly vary, which means that it is even more important to consider various choices that players can take and their direct relationship with tactics and strategy. The player, as an indispensable element of the game, is the person who establishes the internal logic of sports, especially team sports (Lames and McGarry, 2007). Therefore, one of the key considerations when organising a small-sided soccer match is choosing a format that gives the players a sense of freedom which encourages their creativity and hence lets them discover various play possibilities (Kelly and Drust, 2009), while being able to manage the spaces according to their technical, anthropometric and physiological characteristics (Gil et al., 2007; Rampinini et al., 2007) and understand both attacking and defensive play and tactical situations.

The various formats of small-sided soccer considered for this study showed an evolution from the smallest to the biggest. Therefore, we could confirm that there is a development in various small-sided soccer formats (from 5v5 to 9v9) which can be applied and directly transferred to 11v11 football in a final stage (Dellal et al., 2011c; Owen et al., 2011). If an 11-a-side soccer pitch (approx. 100 × 70 meters) is divided into different imaginary spaces (based on lines/positions, pitch areas, duels among players, triangles, etc.), the concept of “reduced spaces” can also be applied to soccer played in these dimensions for a large number of moves.
